# A 20-Year Follow-Up Study of Objectively Measured Physical Activity

**DOI:** 10.3390/ijerph18063076

**Published:** 2021-03-17

**Authors:** Anders Raustorp, Andreas Fröberg

**Affiliations:** Department of Food and Nutrition, and Sport Science, University of Gothenburg, 405 30 Gothenburg, Sweden; anders.raustorp@gu.se

**Keywords:** health behaviour, pedometry, public health, tracking

## Abstract

Background: The objectives of this study were to explore the effect of time, long-term tracking, and the proportion of objectively measured physical activity (PA) from early adolescence to the mid-thirties. Methods: PA was measured as mean steps per day (SPD) with pedometers during 2000 (T1), 2003 (T2), 2005 (T3), 2010 (T4), 2016 (T5) and 2020 (T6). Data from 64 participants (*n* = 32 males) were analysed from their early adolescence (T1) to their mid-thirties (T6). Results: SPD decreased in the total sample and among males and females (all, *p* < 0.001). Males took more mean SPD than females during T1 (*p* = 0.002), whereas females took more mean SPD during T2 (*p* = 0.009) and T6 (*p* = 0.008). Males’ mean SPD tracked between T1 and T2 (*p* = 0.021), T2 and T3 (*p* = 0.030), T3 and T4 (*p* = 0.015) and T4 and T5 (*p* = 0.003). Females’ mean SPD tracked between T3 and T4 (*p* = 0.024) and T5 and T6 (*p* < 0.001). In the total sample, more mean SPD were found on weekdays compared to weekend days at T3 (*p* = 0.017) and T5 (*p* < 0.001). Conclusions: SPD decreased between T1 and T6. Mean SPD tracked low-to-moderate in the short time span. From late adolescence to the mid-thirties, more mean SPD was observed during weekdays compared to weekend days.

## 1. Introduction

There is compelling evidence to suggest positive health effects of regular physical activity (PA) among children, adolescents, and adults [1]. It is therefore critical that about 80% of children and adolescents, and about 30% of adults do not reach contemporary PA recommendations [2]. Of particular interest for PA promotion is the transition from adolescence to adulthood. Early adulthood has been reported to be critical to establish lifestyle behaviours [3] since many physiological and psychological changes occur that might influence PA [4] and manifest differently in males compared to females [3]. To date, interventions have been conducted at different domains, such as active transportation, work-site, leisure time, and household activities [5]. In addition, the opportunity to increase PA during weekend days has gained increased interest. Research suggests that children and adolescents [6,7], as well as adults [8], are more active on weekdays compared to weekend days. A limitation, however, is that previous research investigating the proportion of PA during weekdays and weekend days is largely cross-sectional. 

Moreover, the promotion of PA through interventions is partly based on the perception that PA tracks over time. Tracking usually refers to the stability over time and denotes the tendency for individuals to maintain their rank or position within a group over time [9]. To investigate tracking, a minimum of two measurement points from the same individuals are required [10]. Research to date demonstrates that PA usually tracks low-to-moderately across different stages of life [9,11,12]. In a recent review including several longitudinal approaches, Hayes et al. found that PA tracked low-to-moderately during the transition from adolescence to young adulthood [13]. Another review, with a focus on obesity-related behaviours, among them physical activity, has also shown that PA tracks stronger for males than females and that tracking weakens with increased length of follow-up [11].

To date, most studies have investigated tracking of PA based on self-reporting [9,11,12]. This is an important limitation since self-reported PA data is prone to recall bias and misreporting [14]. The introduction of activity monitors, however, has improved the ability to measure PA during the last decades. Many activity monitors, such as accelerometers and pedometers, are convenient, unobtrusive and relatively unbiased [15]. However, several issues exist regarding appropriate data reduction and standardisation when using accelerometers. Furthermore, given that identical instruments should be used when investigating tracking of PA, another issue might be the recurrent introduction of new generations of accelerometers. As an alternative to accelerometers, pedometers provide valid and reliable estimates of PA in terms of steps per day (SPD) [16,17].

Since most available studies are based on self-reporting [9,11,12], studies that investigate tracking of PA over time by means of activity monitors will increase our knowledge within the field. As far as we know, no study to date has explored tracking of PA during a 20-year time period by means of activity monitors. The aim of this study was three-fold. The first aim was to explore the effect of time on pedometer-determined SPD. The second aim was to explore long-term tracking of pedometer-determined SPD. The third aim was to explore the proportion of SPD during weekday and weekend days from adolescence into adulthood. Our three hypotheses were: (i) there is an effect of time on pedometer-determined SPD; (ii) the tracking of pedometer-determined SPD will be lower, the longer the follow-up; and (iii) weekday pedometer-determined SPD will be higher than that of the weekend day.

## 2. Materials and Methods

### 2.1. Study Design and Sample

This 20-year follow-up study of pedometer-determined SPD begun with the first data collection during 2000. Data for SPD, body height and body weight were collected from a convenience sample comprising 289 adolescents aged 12–14 years in three middle-class communities in the south east of Sweden [18,19]. Three years later, in 2003, a second data-collection was conducted in the same geographical area among adolescents aged 15–17 years (*n* = 375) of which 93 participated 3 years earlier [20]. This group formed a follow-up group that was further invited to participate in data collections in October 2005 (aged 17–19 years), 2010 (aged 22–24 years), 2016 (aged 28–30 years) and 2020 (aged 32–34 years). Six measurement points in total were thus included in the present study: Time 1 (T1) (2000), Time 2 (T2) (2003), Time 3 (T3) (2005), Time 4 (T4) (2010), Time 5 (T5) (2016), and Time 6 (T6) (2020). During these 6 measurement points, reasons not to participate were lack of interest (T1–T6) or inability to track the individuals (T4–T6). Participants with data from at least 4 measurement points of which at least 1 in adulthood (T4–T6) were included in the study.

### 2.2. Procedure and Equipment

During all 6 measurement points, the criterion pedometer Yamax™ (SW-200 Tokyo, Japan) [21] was used to collect data for SPD. The participants were provided with the following instructions at T1–T6: attach the pedometers to the waistband (placed in line with the midpoint of the right knee) and wear the pedometers during the whole day. This meant that they should put on the pedometer after dressing in the morning and wear it until bedtime. During T1–T3, data for SPD was collected during school-hours where researchers (a) collected the pedometers; (b) documented SPD; and (c) resealed the pedometers and returned them to the participants. This procedure was carried out each day during a 24-h interval. During T4–T6, however, the participants had graduated from school. Therefore, the participants were provided with pedometers via mail, and data for SPD were self-recorded and self-reported. During T1–T6, a brief survey was used to identify compliers and non-compliers. The participants completed the survey to verify that the pedometer had been worn properly during the previous day. During all 6 measurement points, data collection took place in October around the time of the autumn equinoxes to control for the potential effect of daylight. 

### 2.3. Anthropometry 

During T1, baseline measurements were collected for body height and body mass. Body height was measured on a tape attached to a wall (Friedrich Richter; Kirchenlaibach, Germany) and rounded down to the nearest centimetre. Body mass was measured on step-up scales (EKS International (Wittiesheim, France) (HEFA Digital AB Halmstad, Sweden) and rounded up to the nearest kilogram. Data for body height and body mass was used to calculate body mass index (BMI).

### 2.4. Data Preparation and Statistical Analysis

Total steps collected during weekdays (T1–T6) were summarised and calculated as mean SPD. During T3–T6, mean SPD during weekend days were also available. To be included in the analysis, steps had to be collected during at least 3 weekdays (at least 1 weekend day during T3–T6) with more than 10 hours per day (and <1 h of non-wear time). As previously recommended [22], days with self-reported values of <1000 or >30,000 steps were excluded from further analysis. 

During the 6 measurement points, some participants had incomplete data. If data from T4, T5 or T6 were missing, imputation was conducted [23] in two different ways. The first way was used when exploring the effect of time on pedometer-determined SPD (aim 1) and the proportion of SPD during weekday and weekend days from adolescence into adulthood (aim 3). To account for the decrease in PA over time, which is commonly observed when comparing different age-groups [24], we here imputed data where the last observation was multiplied with the ‘percent mean group change’ between T3 and T4; T4 and T5; and T5 and T6, respectively. The second way was used when exploring long-term tracking of pedometer-determined SPD (aim 2). Here, missing values were replaced by the ‘mean of the group’ at T4, T5 and T6. Imputation was made in 9 (*n* = 6 males) participants during T4, in 28 (*n* = 15 males) participants during T5 and in 23 (*n* = 11 males) during T6. The present study included 64 individuals (*n* = 32 males) with data (complete data and/or imputed) from T1–T6 which represents 22% of the baseline sample (T1) and 69% of the first follow-up sample formed in 2003 (T2). 

Means and standard deviations (SD±) were calculated for age and mean SPD. All data analyses were performed with SPSS (IBM, Armonk, NY, USA) with the p-value set at 0.05. The effect of time on mean SPD (differences between the 6 measurement points) was analysed with repeated ANOVA measures (rANOVA). Furthermore, sex-differences in mean SPD at T1–T6 were analysed with independent *t*-test. Long-term tracking of mean SPD across the 6 measurement points was analysed with Pearson product-moment correlation (r), and interpreted as low (<0.30), moderate (0.30–0.59), and reasonably good tracking (≥0.60) [9]. Differences in proportions of mean SPD during weekdays and weekend days from adolescence (T3, 2005) to adulthood (T4–T6, 2010–2020) were analysed with paired samples *t*-test.

### 2.5. Ethics

Written informed consent was obtained from the schools, parents (T1–T3) and the participants (T1–T6) prior to each data collection period. During the 6 measurement points, the participants could withdraw their participation at any time without providing any further explanation. 

## 3. Results

### 3.1. Sample Characteristics 

At T1 (baseline), males’ and females’ mean-age were 12.9 (±0.9 years) and 12.9 (±0.8 years), respectively. Mean body height were 1.62 (±0.1 m) and 1.60 (±0.07 m) among males and females, respectively. The corresponding figures for mean body mass were 51.6 (±9.8 kg) among males and 52.8 (±11.7 kg) among females. The percentage classified as overweight/obese at baseline were 3.4% and 11.5% among males and females, respectively. No differences were observed for BMI and mean SPD when comparing the T1 and T6 sample at baseline among males and females, respectively. 

### 3.2. Differences in Steps per Days between the Six Measurement Points

**Figure 1:**
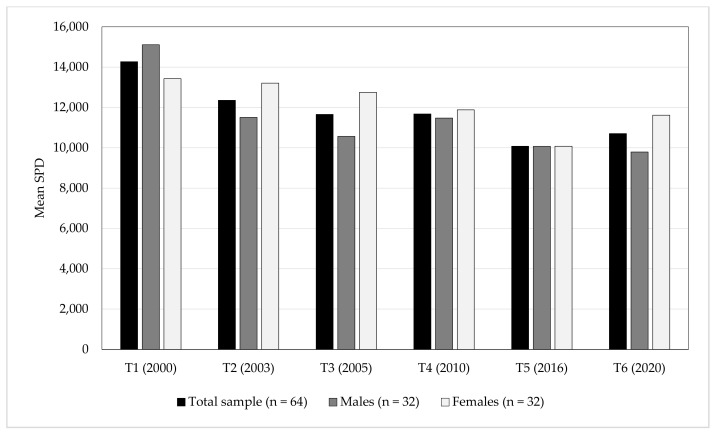
Mean SPD at T1–T6 among the total sample (black bars), and stratified by males (dark grey bars) and females (light grey bars), respectively.

Table 1 summarises descriptive data across the 6 measurement points. There was an effect of time on mean SPD in the total sample (*p* < 0.001, effect size = 0.58), as well as for males (*p* < 0.001, effect size = 0.74) and females (*p* < 0.001, effect size = 0.57). During T1, males took more mean SPD than females (*p* = 0.002), whereas females took more mean SPD than males during T2 (*p* = 0.009) and T6 (*p* = 0.008). Figure 1 show mean SPD at the 6 measurement points among the total sample, and stratified by males and females, respectively.

### 3.3. Tracking of Steps per Day across the Six Measurement Points

The tracking (correlation) of mean SPD across the six measurement points are presented in Table 2. In the total sample, the correlations were significant between T2 and T3 (*r* = 0.31, *p* = 0.012), T3 and T4 (*r* = 0.40, *p* = 0.01), T4 and T5 (*r* = 0.41, *p* = 0.01), and T5 and T6 (*r* = 0.51, *p* < 0.001). Among males, the correlations were significant between T1 and T2 (*r* = 0.41, *p* = 0.021), T2 and T3 (*r* = 0.38, *p* = 0.030), T3 and T4 (*r* = 0.43, *p* = 0.015), and T4 and T5 (*r* = 0.50, *p* = 0.003). Among females, the correlation was significant between T3 and T4 (*r* = 0.40, *p* = 0.024), and T5 and T6 (*r* = 0.63, *p* < 0.001).

### 3.4. Steps per Day during Weekdays and Weekend Days from Adolescence to Adulthood

Table 3 presents mean SPD during weekdays and weekend days from adolescence (T3) to adulthood (T4–T6). In the total sample, significantly more mean SPD on weekdays compared to weekend days were found at T3 (*p* = 0.017) and T5 (*p* < 0.001). Among males, significantly more mean SPD were shown on weekdays compared to weekend days at T5 (*p* < 0.001). At T3, T4, and T6, however, the results did not show significantly more mean SPD during weekdays. Among females, significantly more mean SPD were shown on weekdays at T5 (*p* = 0.013). The results also did not show significantly more mean SPD during weekdays at T3 and T6. Furthermore, among females, the results did not show significantly more mean SPD during weekend days than weekdays at T4.

## 4. Discussion

The main finding of this study was the significant effect of time where SPD decreased between the 6 measurement points. As previously reported [20], a decrease in mean SPD was observed between T1 and T2 among males, which is consistent with self-reported longitudinal data from Finland [25] and the Netherlands [26]. The significant decrease in mean SPD observed during early adolescence among males appears, according to our data, to continue during early adulthood into the mid-thirties. In the first decade (from age 12–14 to 22–24 years), the decrease in mean SPD was 25% compared to 15% during the second decade (from age 22–24 to 32–34 years). Compared to males, mean SPD decreased to a much lesser extent among females with a decrease of 12% and 2% during the first and second decade, respectively.

A previous review estimated that mean SPD for youth males and females generally were 12,000–16,000 and 10,000–13,000, respectively [27]. Interestingly, the results in this study showed that males on average took fewer mean SPD than females from age 15 to 34 years, which is somewhat in contrast to previous research [27]. Although differences are noted across countries, peak-values of mean SPD generally occur before age 12 and then gradually decrease to approximately 8000–9000 SPD at age 18 [27]. Tudor-Locke and Myers [28] reported a span of 7000–13,000 mean SPD in young adults aged 20–50. There is currently a lack of Swedish representative data of pedometer-determined mean SPD among adults. In a study with representative data collected in Denmark, Mathiessen et al. [29] reported mean SPD of 8500 and 8000 during adulthood (mean age of 49 years) for males and females, respectively. In our sample, mean SPD during adulthood (mean age of 32.9 years) are higher (i.e., 9792 SPD among males and 11,618 SPD among females) than in the neighbour country Denmark. It may be argued that the process of collecting data, for many days, at 6 different measurement points from early adolescence to the thirties is an intervention per se. The effectiveness of pedometer interventions on SPD have previously been reported [5], and a three-week intervention has been shown to significantly impact SPD as long as four years after the intervention [30].

As partly reported in previous research [20,31,32], low-to-moderate tracking of mean SPD was observed in the shorter time span (e.g., T1–T2 and T4–T5) with higher correlation among males than females during early adolescence. The strongest correlation, judged as reasonably good, was seen among females between T5–T6. Although significant, the strength of the tracking indicates difficulties to foresee activity levels years ahead based on activity levels at earlier years. 

Moreover, some of these findings correspond to international studies using primary self-reported data [11]. Based on these results, it appears that mean SPD tracks low-to-moderate even when using activity monitors. We found no tracking between T1 and T6 among both males and females. Thus, we could not confirm earlier findings suggesting significant tracking from early adolescence to adulthood [25], and this may be explained by the different measurement methods used (activity monitors vs. self-report). 

A recent Scandinavian study, using cross-sectional data, observed that both adolescents and adult are more physically active during weekdays compared to weekend days [8], and pointed out the need of longitudinal studies to confirm these findings. When we follow the same individuals over a period of 15 years (from T3 to T6, mean ages 15–17 to 32–34 years) we can confirm these earlier suggestions for males. At T3–T6, males engaged in more PA than females on weekdays. The decrease in PA on weekend days varied from 21% at T5 to 6% at T6. In addition, females engaged in more PA on weekdays compared to weekend days and the decrease varied from 13% at T3 to 7% at T6. The only exception was at T5 (mean age 22.9 years) when females engaged in more PA on weekend days. This study supports the idea of tailoring interventions to increase PA during weekends, especially among males. 

### Strengths and Limitations

The strength of this study is that identical instruments and study protocols for data collection were used during T1–T6. A limitation might be the sample, which was enrolled in overall geographically narrowed mid-socioeconomic communities, suggesting that caution is needed for generalisation. Although the follow-up group did not differ in terms of mean SPD, relative to the baseline sample, the limited number of participants in this 20-year follow-up study is also acknowledged as a significant limitation. Factors such as age at baseline, biological variation, socioeconomic variation, major environmental changes and measurement variability have been reported to influence inter-age correlations [33]. These potential covariates remain uncontrolled in this study. Even though data was collected in October during all 6 measurement points, with roughly 12-hours of daylight in Scandinavia, different weather conditions across the data collection periods may also have influenced the result.

An identical model of Yamax™ (SW-200 Tokyo, Japan) [21] pedometer was employed during all 6 measurement points. However, while the pedometers were sealed and SPD documented by the researchers during T1–T3, SPD were self-recorded and self-reported by the use of mailed (unsealed) pedometers during T4–T6. These 2 different approaches are not fully comparable. Because self-reported measurements of PA have been called into question due to recall bias and misreporting [14], we cannot rule out that similar biases are present when self-recording and self-reporting SPD.

As we focused on SPD, the Yamax™ pedometer is a reasonable choice of activity monitor although they do not measure activities such as biking, swimming and weight-bearing PA. Another shortcoming is that Yamax™, by using the mechanism of a spring suspended lever, does not provide immediate information about the PA intensity. Participants who engaged in PA characterised by high intensity/low duration may have collected few SPD and, as a result, been judged as limited activity by the pedometer [34]. In this regard, the accelerometer would have been an alternative activity monitor. During T1, however, the accelerometer research was in its infancy, and across the time span of this study, new generations of accelerometers have been developed [35], different epoch durations have been recommended, and the uncertainty regarding optimal guidelines to handle collected data (e.g., cut-points for PA intensity [36]) may be a burden when conducting long-term follow-up studies.

Imputation is a common way to avoid missing data [23] and we chose to impute some missing values in this dataset. The decision to impute data in two different ways was because the three aims would be affected differently. To replace missing data with “group mean” does not take into account the decline in PA, which is observed when comparing different age groups [24]. To explore the consequences of imputing “group mean”, we therefore also replaced missing data with “mean group change” which, in this case, gave significant correlation observed between T4 and T5, and T5 and T6. Given this discrepancy between the two different ways of imputation, we urge for caution when interpreting the correlation between T4 and T5, and T5 and T6. 

Finally, T6 was conducted in October 2020 when COVID-19 restrictions, such as work from home, social distancing and restrictions regarding exercise facilities, were prevalent in Sweden. This might have affected the level of PA during the week of measurement. In comparison to other countries, Sweden has had no total lockdown to this date. It has been reported that this strategy might have affected the physical activity, measured as SPD, to a lesser extent in Sweden (Stockholm) than in other countries [37]. COVID-19 might have affected the level of PA during weekdays more than during weekend days; as an example, work from home might decrease the weekday active transportation to and from work.

## 5. Conclusions

There was a significant effect of time where SPD decreased between the six measurement points. In this geographically narrowed and size-limited sample, SPD tracked low-to-moderate in the short time span yet tracked non-significantly from early adolescence to the mid-thirties. These findings indicate difficulties to foresee activity levels years ahead based on activity levels at earlier stages of life. More mean SPD was observed during weekdays compared to weekend days, which supports the idea of tailoring interventions to increase PA during weekend days, especially among males. 

## Figures and Tables

**Table 1:** Descriptive data for participants (numbers and age), and mean (SD ±) steps per day (SPD) at T1 (2000), T2 (2003), T3 (2005), T4 (2010), T5 (2016), and T6 (2020) in the total sample, and stratified by males and females, respectively.

Time	Total Sample (*n* = 64) *			Malese (*n* = 32) **			Femalese (*n* = 32) ***	
	Age (y)	SPD (SD±)		Age (y)	SPD (SD ±)		Age (y)	SPD (SD±)
T1	12.9	14,271 (± 2876)		12.9	15,114 (± 2933)		12.9	13,427 (± 2595)
T2	15.9	12,356 (± 2790)		15.9	11,507 (± 2926)		15.9	13,206 (± 2403)
T3	17.9	11,658 (± 3142)		17.9	10,567 (± 2300)		17.9	12,749 (± 3509)
T4	22.9	11,680 (± 3391)		22.9	11,474 (± 3670)		22.9	11,885 (± 3132)
T5	28.9	10,076 (± 2483)		28.9	10,075 (± 2712)		28.9	10,076 (± 2295)
T6	32.9	10,705 (±2787)		32.9	9792 (± 2542)		32.9	11,618 (± 2759)

Repeated measures ANOVA was conducted to explore the effect of time. * Significant effect for time (Wilk´s Lambda = 0.42, F(5, 59) = 15,97, *p* < 0.001;Multivariate eta^2^ = 0.57) ** Significant effect for time (Wilk´s Lambda = 0.26, F(5, 27) = 15,53, *p* < 0.001; Multivariate eta^2^ = 0.74) *** Significant effect for time (Wilk´s Lambda = 0.43, F(5, 27) = 7,15, *p* < 0.001, Multivariate eta^2^ = 0.57).

**Table 2:** Correlations (*r*) matrix for mean SPD at T1 (2000), T2 (2003), T3 (2005), T4 (2010), T5 (2016), and T6 (2020) in the total sample and stratified by males and females, respectively.

**Total Sample (*n* = 64)**						
	T1	T2	T3	T4 ^a^	T5 ^a^	T6 ^a^
T1						
T2	0.18					
T3	−0.01	0.31 *				
T4 ^a^	−0.03	−0.00	0.40 **			
T5^a^	0.03	−0.00	0.01	0.41 **		
T6^a^	−0.10	−0.02	−0.02	0.21	0.51 **	
**Males (*n* = 32)**						
	T1	T2	T3	T4 ^a^	T5 ^a^	T6 ^a^
T1						
T2	0.41 *					
T3	0.38 *	0.32				
T4 ^a^	0.13	−0.05	0.43 *			
T5 ^a^	0.15	−0.04	0.32	0.50 **		
T6 ^a^	−0.09	−0.15	0.19	0.33	0.34	
**Females (*n* = 32)**						
	T1	T2	T3	T4 ^a^	T5 ^a^	T6 ^a^
T1						
T2	0.15					
T3	−0.09	0.18				
T4 ^a^	−0.16	−0.13	0.40 *			
T5 ^a^	0.00	−0.06	−0.26	0.31		
T6 ^a^	0.01	0.04	−0.35	−0.06	0.63 **	

^a^ Imputed data. * Correlation is significant at the 0.05 level (2-tailed). ** Correlation is significant at the 0.01 level (2-tailed).

**Table 3:** The proportions of mean SPD during weekdays and weekend days from adolescence (T3, 2005) to adulthood (T4–T6, 2010–2020) in the total sample, and stratified by males and females, respectively.

	Total Samplee (*n* = 64)			Malese (*n* = 32)			Femalese (*n* = 32)		
	SPD		*p*	SPD		*p*	SPD		*p*
	Weekdays	Weekend Days		Weekdays	Weekend Days		Weekdays	Weekend Days	
T3	11,658	10,320	0.017	10,567	9484	0.096	12,749	11,156	0.084
T4	11,680	11,295	0.402	11,475	10,418	0.126	11,885	12,173	0.636
T5	10,076	8612	<0.001	10,076	7993	<0.001	10,077	9230	0.013
T6	10,705	10,064	0.105	9792	9249	0.325	11,619	10,879	0.202

## Data Availability

The data presented in this study are available on request from the corresponding author. The data are not publicly available due to restrictions in ethical approval.
